# High Expression of NEK2 and PIM1, but Not PIM3, Is Linked to an Aggressive Phenotype of Bronchopulmonary Neuroendocrine Neoplasms

**DOI:** 10.1007/s12022-020-09629-y

**Published:** 2020-06-05

**Authors:** Ewelina Motylewska, Marcin Braun, Henryk Stępień

**Affiliations:** 1 Department of Immunoendocrinology, Chair of Endocrinology, Medical University of Lodz, Pomorska 251, 92-213 Lodz, Poland; 2 Department of Pathology, Chair of Oncology, Medical University of Lodz, Pomorska 251, 92-213 Lodz, Poland

**Keywords:** Bronchopulmonary neuroendocrine neoplasm, Expression, NEK2, PIM1, PIM3

## Abstract

Dysregulations of the NEK2 and PIM1-3 kinase signaling axes have been implicated in the pathogenesis of several cancers, including those with a neuroendocrine phenotype. However, their impact on bronchopulmonary neuroendocrine neoplasms (BP-NENs) has not been investigated. The aim of this pilot study was to determine mRNA and protein levels of NEK2, PIM1, and PIM3 in a group of 49 patients with BP-NENs: 11 typical carcinoids, 5 atypical carcinoids, 11 large cell neuroendocrine carcinomas, 22 small cell lung carcinomas (SCLC). The expression was measured using TaqMan-based RT-PCR and immunohistochemistry. NEK2 and PIM1 mRNA levels were higher in the SCLC patients than in the other BP-NEN groups (*p* < 0.001). There was an association between NEK2 mRNA and protein expression (*p* = 0.023) and elevated NEK2 mRNA levels were related to reduced survival in BP-NEN patients (*p* = 0.015). Patients with higher PIM1 protein expression had also diminished survival comparing with those with weak or no PIM1 expression (*p* = 0.037). Elevated NEK2 and PIM1 expression were related to aggressive tumor phenotype and indirectly affected the overall survival of BP-NEN patients. Our pilot study supports the need for future investigation of the biological function of NEK2 and PIM1 in BP-NEN transformation to verify the clinical value of our findings.

## Introduction

Neuroendocrine neoplasms (NENs) are rare malignancies arising from cells throughout the diffuse endocrine system, and their incidence has significantly risen in the last years. NENs comprise a heterogeneous group of neoplasms with a wide spectrum of clinical behavior depending on tumor localization and the presence of hormonal hypersecretion. According to the 2015 World Health Organization (WHO) classification, bronchopulmonary neuroendocrine neoplasms (BP-NEN) comprise four tumor entities which differ with respect to their histology, mitotic count, and extent of necrosis: typical carcinoids (TC), atypical carcinoids (AC), small cell lung cancers (SCLC), and large cell neuroendocrine carcinomas (LCNEC) [1]. TC and AC represent the well-differentiated forms of BP-NENs named neuroendocrine tumors (NETs), while SCLC and LCNEC comprise poorly differentiated lung NENs called neuroendocrine carcinomas (NECs) [2]. The differences in the biology and clinical course of NET and NEC tumors entail distinct therapeutic approaches. Unlike lung NECs, which are usually treated with chemotherapy, advanced stages of lung NETs can be managed with several different therapies (somatostatin analogs, the mTOR inhibitor everolimus, chemotherapy, and peptide receptor radionuclide therapy). Many different targeted therapies have been investigated with limited or no results, and others are currently still under investigation for treating lung NETs [3, 4] and NECs [5–7]. In particular, new therapeutic strategies are needed to change the natural history of highly aggressive SCLC, as the effectiveness of chemotherapy has plateaued [5–7]. The lack of relevant new therapeutic approaches in SCLC is in prominent contrast to personalized medicine for advanced stage non-small cell lung cancers (NSCLC), which benefits from targeted therapies and immunotherapy [8].

Protein kinases regulate many cellular signaling pathways, and their disruption is often associated with carcinogenesis [9]. Although dysregulations of the NEK2, PIM1, and PIM3 kinase signaling axes have been implicated in the pathogenesis of several cancers [10, 11], including those with a neuroendocrine phenotype [12, 13], their role in the pathogenesis of BP-NENs has not been determined.

NEK2 is a serine/threonine kinase belonging to the NIMA (never in mitosis gene A)-related family, involved in cell division and cell cycle regulation by centrosome splitting. With the alternate splicing, NEK2 is expressed as three splice variants, namely NEK2A, NEK2B, and NEK2C. NEK2A is the full-length protein, with 445 amino acids (48 kDa), and is the most studied variant [10]. Aberrant NEK2 expression and activity lead to dysregulation of the centrosome cycle and aneuploidy [14]. Previous studies have found NEK2 to play roles in chromosome instability, tumorigenesis, cancer progression, and drug resistance [15–17]. Accumulating evidence have shown that mRNA and/or protein level of NEK2 is upregulated in primary tumor tissues or cancer cell lines of several cancers [10], i.e., in pancreatic neuroendocrine tumors [12].

The human PIM kinase is a highly conserved serine-threonine protein kinase named after the proviral integration site for MuLV, the genomic site where it was discovered. The PIM family, composed of three isoforms, viz. PIM1-3, plays a key role in the control of cell proliferation, survival, and migration [11].

PIM1 kinase is a constitutively active downstream effector molecule of many cytokine signaling pathways controlled by JAK/STAT (Janus kinase/signal transducer and activator of transcription) transcription factors. PIM1 activity promotes tumor cell growth and survival through the modification of several cell cycle regulators and apoptosis mediators. PIM1 kinase can contribute to tumorigenesis also by enhancing MYC-regulated oncogenic signaling pathways [18]. Moreover, PIM1 has been found to be associated with drug resistance of cancer cells [11, 19]. PIM1 is mainly expressed in the thymus, spleen, bone marrow, fetal liver, and other hematopoietic organs, while its expression is absent in adult tissues [11, 20]. Abnormal expression of PIM1 has been linked to hematological malignancies [20, 21], colon [22], bladder [23], head and neck squamous cell carcinoma [24], and prostate cancer [25, 26], including the neuroendocrine variant: one of its most aggressive forms [13].

PIM3 suppresses apoptosis and promotes cell growth and survival, thereby enhancing cell proliferation of normal and malignant cells. PIM3 is expressed in various tissues, including the heart, brain, lung, kidney, spleen, placenta, skeletal muscle, and peripheral blood leukocytes. PIM3 protein is barely detected in normal adult endoderm-derived organs such as the liver, pancreas, colon, and stomach [27, 28]; however, its expression is augmented in malignant lesions of these organs [27, 29–33]. While PIM1 and PIM2 levels are mostly elevated in hematologic malignancies and prostate cancer, increased PIM3 expression is typically observed in other solid tumors [34].

Therefore, the aim of this pilot, clinical-based study was to verify a possible link between NEK2, PIM1, PIM3, and BP-NENs in the context of known prognostic features.

## Materials and Methods

### Study Cohort

A total of 60 formalin-fixed paraffin-embedded tumor blocks (FFPEs) from 49 patients (27 males, 22 females) with a median age of 65 years (60.00–70.00) were provided by Department of Pathology, Chair of Oncology, Medical University of Lodz, Poland. All patients recruited to the study had been newly diagnosed with BP-NENs from 2008 to 2019: 11 patients were diagnosed with TC, 5 with AC, 22 with SCLC, and 11 with LCNEC. All tumors were histopathologically examined and classified according to the WHO 2004 or 2015 classification of lung neoplasms [35, 36]. For statistical analysis, all patients were further divided into two groups with NETs (*n* = 16) and NECs (*n* = 33).

The sample material comprised either resected specimens, for NETs, or biopsy specimens, for NECs. The primary tumor samples were divided into two or three parts, depending on tumor size, and embedded in separate paraffin blocks. The number of samples used in the study varied from one to two FFPEs per patient.

Ethics committee approval was obtained from the Institutional Board of the Medical University of Lodz (Number RNN/145/18/KE).

### Total RNA Isolation

Total RNA was extracted from FFPE tissue using the miRNeasy FFPE Kit (Qiagen). In brief, FFPE slices were processed in 1.5-mL Eppendorf tubes, deparaffinized with 160 μl deparaffinization solution, and then digested with proteinase K and DNase I. Purification of extracted total RNA was performed with RNeasy MinnElute columns according to the manufacturer’s instructions. The yield and quality (the ratio of absorptions at 260/280 nm) of RNA product were measured using PicoDrop spectrophotometer (Picodrop Limited, UK). The purified total RNA was immediately used for cDNA synthesis or stored at − 80 °C until use.

Forty-five patients with good quality total RNA were taken for further analysis.

### NEK2, PIM1, and PIM3 mRNA Expression

cDNA was generated with the Maxima First Strand cDNA Synthesis kit for RT-qPCR (ThermoFisher) according to the manufacturer’s protocol. Briefly, 500 ng of total RNA was used as starting material, to which the reaction components and reverse transcription master mix were added. The reaction proceeded for 10 min at 25 °C followed by 15 min at 50 °C. After inactivation of Maxima Reverse Transcriptase (5 min at 85 °C), the cDNA samples were kept frozen at − 20 C.

Measurement of mRNA expression was done using standard TaqMan® Gene Expression Assays (Applied Biosystems): Pim-3 Proto-Oncogene, Serine/Threonine Kinase (PIM3, Hs00420511_ g1), Pim-1 Proto-Oncogene, Serine/Threonine Kinase (PIM1, Hs01065498_ m1), NIMA Related Kinase 2 (NEK2, Hs00601227_mH), and actin beta (ACTB, Hs 01060665_ g1) as the endogenous control. TaqMan PCR assays were performed in 10**-**μL reactions included 50 ng cDNA, 5-μL TaqMan™ Fast Advanced Master Mix (ThermoFisher), and 0.5-μL appropriate TaqMan Gene Expression Assay. All reactions were run in duplicate on a 7900 HT Fast Real-Time PCR System (Applied Biosystems). The following thermal cycling specifications were performed: 10 min at 95 °C and 40 cycles each for 10 s at 95 °C and 60 s at 60 °C. All reactions were run in duplicate.

Kinase expression levels were calculated using the RQ (the 2^^−ΔΔ*Ct*^ method).

### Immunohistochemistry for NEK2, PIM1, and PIM3 Protein Expression

Immunohistochemical protein expression was studied in 59 FFPE samples using Anti-NEK2 Rabbit polyclonal antibody (Abcam), PIM1 Rabbit Polyclonal Antibody (C-term) (Abgent), and PIM3 Polyclonal Antibody (ThermoFisher Scientific), processed with the EnVision (DAKO) system. Tumor sections were examined for kinase immunoreactivity under a microscope at × 20 and × 40 magnifications. The IHC results were validated using positive and negative tissue controls in all series of immunostained slides. The following positive controls were set up: NEK2 on ovarian carcinoma, PIM1 on breast cancer, and PIM3 on gastric carcinoma. To examine negative control staining, neoplastic tissue slides were evaluated using mouse isotype antibody Ready-to-Use FLEX Negative Control Mouse (Cocktail of mouse IgG1, IgG2a, IgG2b, IgG3 and IgM, IR750, DAKO, Denmark). The tests were carried out using Autostainer Link 48 (Dako, Denmark).

For the semiquantitative immunohistochemical scoring (IRS), we applied a cutoff of 10% immunolabeled cells for BP-NENs. Cytoplasmic staining was considered for the evaluation of NEK2 and PIM3 expression and cytoplasmic and nuclear for PIM1 expression.

Negative NEK2, PIM1, and PIM3 staining in tumor sections were defined as IRS 0. All positive sections were further scored according to three grades of staining intensity (IRS 1, 2, or 3).

Moreover, for further statistical analysis, all sections were additionally divided into a two-point classification: IRS 0 and 1 were placed into one group, and IRS 2 and 3 into a second.

The immunohistochemical stainings were examined in standard light microscopy (Light Microscope BX43, OLYMPUS Europa SE &amp; CO, Hamburg, Germany). The selected sections were scanned and representative images were taken using UltraFast Scanner (Philips IntelliSite Solution, USA) with DigiPath™ Professional Production Software (Xerox, Norwalk, CT, USA).

### Statistical Analysis

Continuous variables are presented as medians followed by IQR and nominal variables are presented as numbers followed by percentages in brackets. The Shapiro-Wilk test was used to determine the distribution. Continuous variables were compared using Student’s *t* test or one-way analysis of variance (ANOVA) in the case of normal distribution and the Mann Whitney *U* test (or ANOVA Kruskal-Wallis) in the case of the non-normal distribution. Differences between categorical variables were evaluated using the *χ*^2^, two-tailed Fisher’s, or Yates exact test. Bonferroni’s correction was used for multiple comparisons. Spearman’s rank test was used for correlation assessment. *p* values < 0.05 were considered statistically significant. The Statistica 13.1 PL package (StatSoft, Tulsa, OK, USA) was used for the analysis.

For the outcome analyses, overall survival was defined as the time period from diagnosis to last follow-up (15th October 2019), with censoring of live patients at the last follow-up. Overall survival data are presented as the Kaplan-Meier survival curves and compared within subgroups using the log-rank test. Cox hazards regression analyses of overall survival adjusted for age were performed for each variable.

## Results

### Molecular Characteristics of the Study Group

**Fig. 1:**
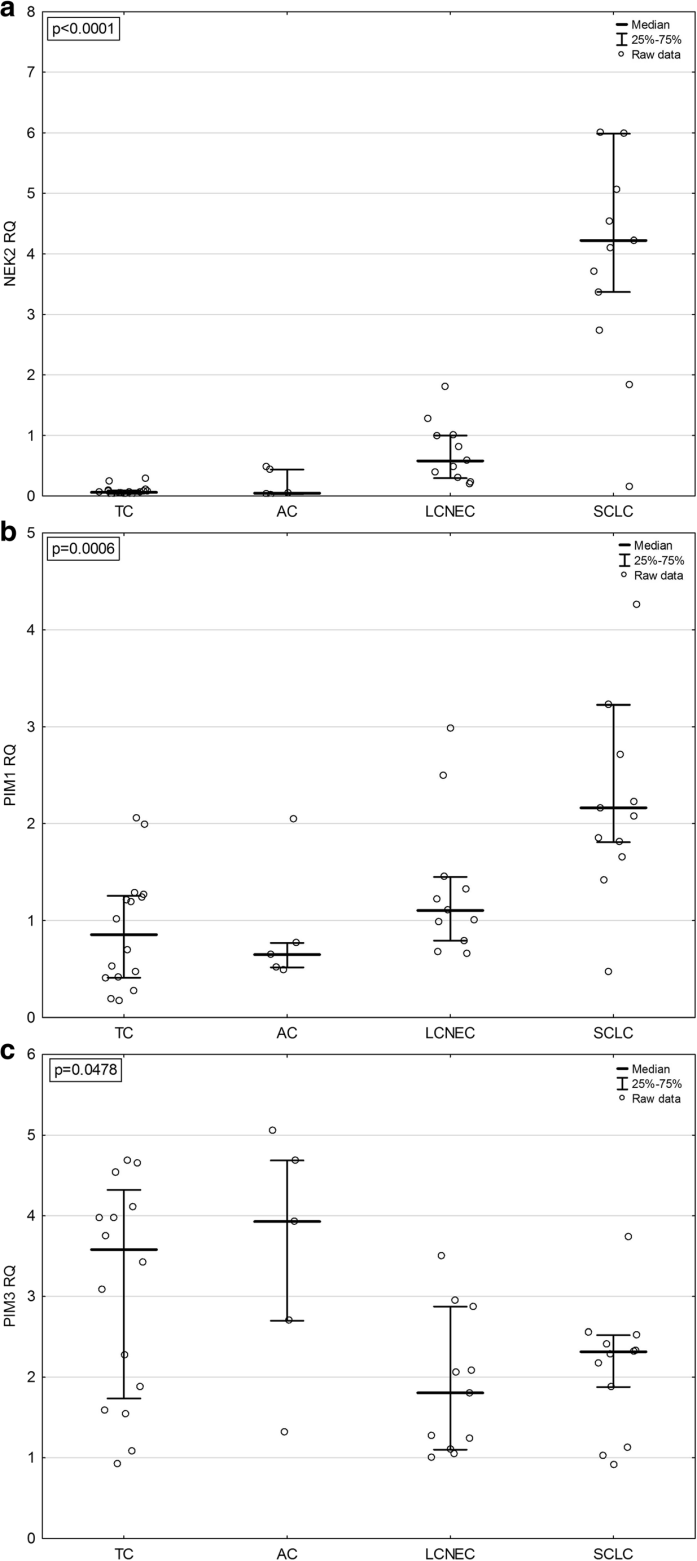
mRNA expression of kinases in different BP-NEN entities: **a** NEK2 expression, **b** PIM1 expression, **c** PIM3 expression. TC, typical carcinoid; AC, atypical carcinoid; SCLC, small cell lung carcinoma; LCNEC, large cell neuroendocrine carcinoma

In the whole BP-NEN group, the highest mRNA level was determined for kinase PIM3 (RQ 2.34 (1.54–3.75)), which was higher than the PIM1 mRNA level (RQ 1.22 (0.66–2.05)). NEK2 mRNA expression in BP-NENs was low (RQ 0.29 (0.07–1.83)). However, NEK2 mRNA levels were significantly increased in SCLC patients (RQ 4.22 (3.37–5.99)) comparing with other entities: TC (RQ 0.06 (0.03–0.09)), AC (RQ 0.05 (0.03–0.44), and LCNEC (RQ 0.58 (0.29–1.00)) (*p* < 0.001) (Fig. 1a). Similarly, mRNA expression of PIM1 was also significantly higher (*p* < 0.001) in SCLC samples (RQ 2.16 (1.81–3.23)) compared with other BP-NEN groups (RQ 0.85 (0.41–1.26), 0.65 (0.52–0.77), and 1.1 (0.79–1.45), for TC, AC, and LCNEC, respectively) (Fig. 1b).

In contrast, PIM3 mRNA levels were significantly higher (*p* = 0.048) in TC and AC (RQ 3.58 (1.74–4.32) and 3.93 (2.70–4.68), respectively) than in the aggressive BP-NEN tumors (RQ 1.81 (1.10–2.87) for LCNEC and RQ 2.32 (1.88–2.52) for SCLC) (Fig. 1c). In addition, PIM1 mRNA expression positively correlated with NEK2 mRNA levels (*p* < 0.05; *R* = 0.63) and the age at diagnosis (*p* = 0.031, *R* = 0.32) in BP-NEN patients. PIM1 mRNA levels were also negatively associated with PIM3 mRNA levels (*p* < 0.05; *R* = − 0.31).

### Immunohistochemical Characteristics of the Study Group

In BP-NEN patients, the most frequent immunoreactivity was observed for PIM3 kinase, being present in 57 (97.0%) samples. Moreover, 25 (42.4%) FFPEs demonstrated strong (IRS 3) PIM3 protein expression. Positive immunoreactivity for NEK2 was detected in 54 (91.5%) BP-NEN sections, and they showed mostly (23 (39.0%) sections) moderate intensity of staining (IRS 2). The least frequent immunoreactivity among all BP-NEN specimens was observed for PIM1 kinase (46 (78.0%) samples) and its expression was mainly weak, with 28 (47.5%) FFPEs characterized by IRS 1.

PIM1 protein expression was slightly, but not significantly, higher in SCLC, as 45.8% of them (11 sections) showed quite intense immunostaining (IRS 2 in 9 (37.5%) and IRS 3 in 2 (8.3%) samples). IRS 3 for PIM1 was not observed in any other BP-NEN groups, and IRS 2 was detected only in 4 (25.0%) TC, 1 (16.7%) AC, and 2 (15.4%) LCNEC.

Similarly, NEK2 protein expression tends to be slightly elevated in SCLC, being present at IRS 2 and 3 in 70.8% of them (17 samples), but only in 50.0% of TC (8 sections), 50% of AC (3 sections), and 61.5% of LCNEC (8 sections). PIM3 protein expression was rather intensive in BP-NENs, as mentioned above, but did not differ significantly between histopathological groups. In addition, a positive correlation was found between NEK2 and PIM1 protein expression in BP-NEN patients (*p* = 0.004).

**Fig. 2:**
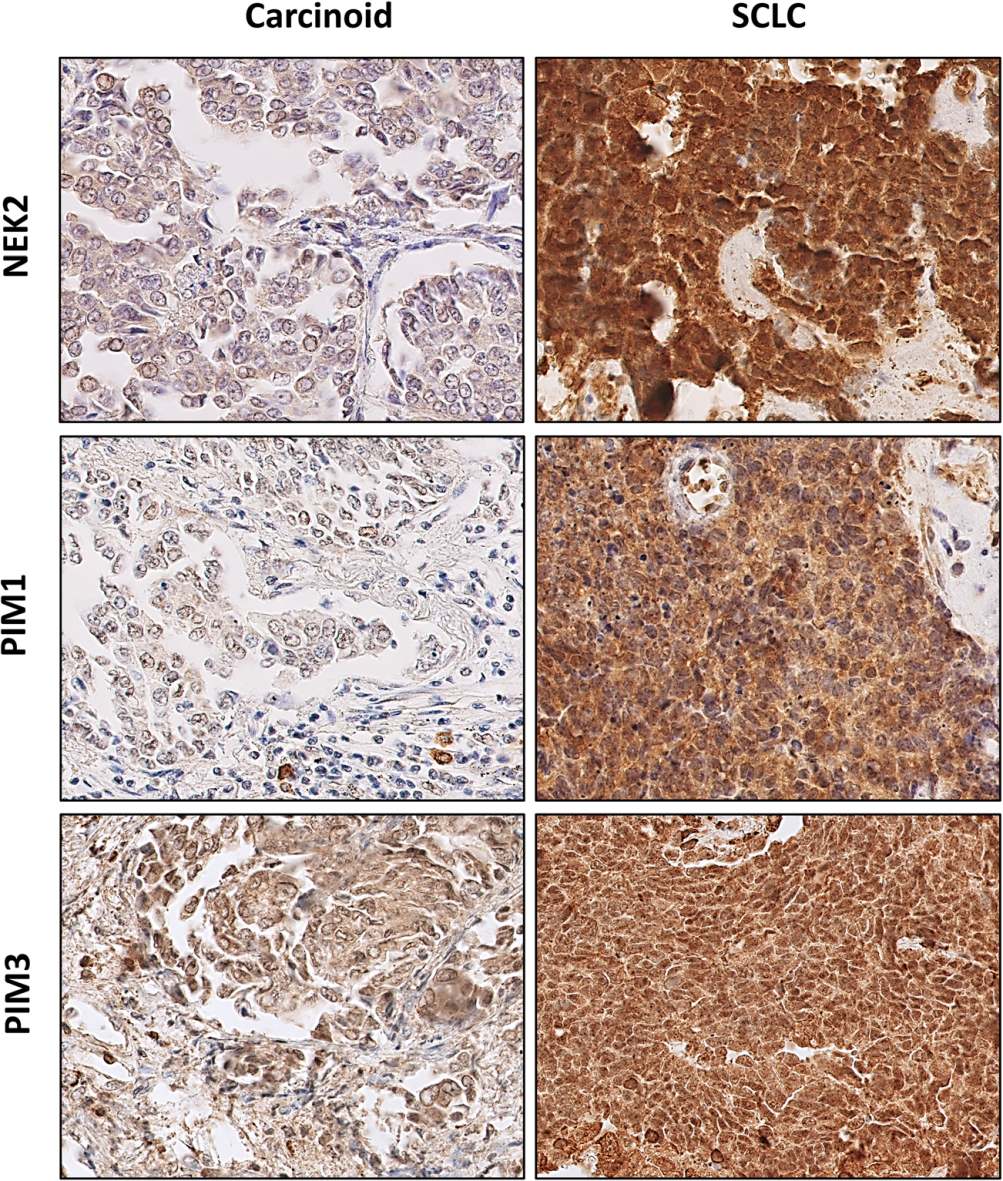
Examples of representative positive immunohistochemical staining for kinases in well-differentiated and poorly differentiated BP-NENs. TC, typical carcinoid; SCLC, small cell lung carcinoma

Immunohistochemical staining for PIM1, PIM3, and NEK2 in different BP-NEN entities is depictured in Fig. 2.

### Association Between mRNA Levels and Protein Expression

An association was found between mRNA and protein levels for NEK2 in BP-NEN patients (*p* = 0.023). No significant associations were found between mRNA and protein levels for kinases PIM1 and PIM3.

### Survival Analyses

**Fig. 3:**
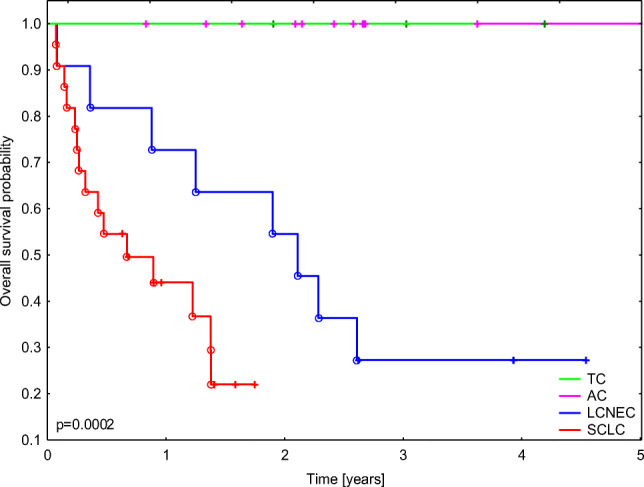
Probability of overall survival in the study group according to diagnosis. TC, typical carcinoid; AC, atypical carcinoid; SCLC, small cell lung carcinoma; LCNEC, large cell neuroendocrine carcinoma

Within the whole BP-NEN group, 23 deaths were observed and the median (IQR) overall survival was 1.4 years (0.1–11.00). Median overall survival (OS, (IQR)) was 2.4 years (1.6–2.7) for TC, 4.2 years (3.0–9.7) for AC, 2.1 years (0.9–2.6) for LCNEC, and 0.7 years (0.3–1.2) for SCLC (Table 1). Significant differences in OS were observed among patients with different BP-NEN types (*p* = 0.0002; Fig. 3). No death cases were recorded in TC and AC groups, and the differences in OS between LCNEC and SCLC patients approached the level of significance (*p* = 0.054). Elevated NEK2 mRNA levels were related to a lower probability of OS in BP-NEN patients (*p* = 0.015; HR = 1.35 (1.06–1.72)). In addition, patients with higher PIM1 protein expression also demonstrated lower OS than those with weak or no PIM1 expression (*p* = 0.037; HR = 4.63 (1.1–19.63)). These observations could be mostly attributed to the relationship between high NEK2/PIM1 levels and an aggressive NEC phenotype. However, higher PIM1 protein expression was shown to be associated with worsened OS also in the subgroup of NEC patients (*p* = 0.045, HR 6.90 (1.05–45.54)).

**Table 1:** Patient characteristics. *TC*, typical carcinoid; *AC*, atypical carcinoid; *LCNEC*, large cell neuroendocrine carcinoma; *SCLC*, small cell lung carcinoma; *IHC*, immunohistochemistry; *RT-PCR*, real-time PCR

Variable	Number (%) or median (IQR)
Sex males/females	27 (55.1)/22 (44.9)
Age at diagnosis (years)	65 (60.0–70.0)
Overall survival (years)	1.4 (0.1–11.0)
TC	2.4 (1.6–2.7)
AC	4.2 (3.0–9.7)
LCNEC	2.1 (0.9–2.6)
SCLC	0.7 (0.3–1.2)
IHC/RT-PCR sample	59 (100.0)/45 (100.0)
TC	16 (27.1)/16 (35.6)
AC	6 (10.2)/5 (11.1)
LCNEC	13 (22.0)/11 (24.4)
SCLC	24 (40.7)/13 (28.9)

As no deaths were observed among NET patients, this subgroup was further studied to obtain recurrence data. Three cases of tumor relapses were observed: one in TC and two in AC. Unfortunately, no statistical analysis was possible due to the small number of events.

## Discussion

The present study is the first to show that NEK2 and PIM1 expression is associated with BP-NEN aggressiveness.

Although previous studies have indicated that the NEK2 gene is involved in lung adenocarcinoma [37, 38] and that non-small cell lung cancers overexpress NEK2 protein [39], its expression in SCLC and other BP-NENs has not been studied so far. However, some papers present NEK2 in the neuroendocrine context. The Cancer Dependency Map Portal reports that SCLC cell lines demonstrate low to moderate dependency on knockout of NEK2 [40]. NEK2 gene expression was also positively associated with high tumor grade of pancreatic neuroendocrine tumors [12]. Our findings in this pilot study indicate that the NEK2 mRNA level is significantly higher in SCLC than in other BP-NENs. NEK2 protein expression, measured by IHC, also appeared to be higher in SCLC than in TC, AC, and LCNEC. This assumption was substantiated by the presence of a statistically significant association between NEK2 mRNA and protein levels in BP-NENs. Moreover, elevated NEK2 mRNA levels were related to reduce OS in BP-NEN patients, and this finding could be explained by an association between high NEK2 expression and an aggressive phenotype. A particularly interesting aspect of our findings is that previous studies based on immunohistochemical and immunofluorescence techniques did not identify NEK2 expression in normal bronchial epithelial cells [39]. These data suggest a possible link between NEK2 overexpression and SCLC development.

Recent studies based on immunohistochemical staining found PIM1 to be highly expressed in NSCLC [41, 42]. Moreover, increased PIM1 levels were associated with worse prognosis [41] and poorer response to chemotherapy in these tumors [42]. PIM1 was also showed to promote highly aggressive, neuroendocrine variant of prostate cancer by epigenetic changes in H19, suggesting it may be implicated in malignant neuroendocrine transformation [13]. Our study for the first time indicated that PIM1 mRNA expression is significantly higher in SCLC samples than in other BP-NEN entities. The immunohistochemical staining results also showed the tendency to augmented PIM1 protein expression in SCLC in comparison with other studied groups. In addition, elevated PIM1 protein expression was found to diminish overall survival throughout the whole BP-NEN group and in the subgroup of NEC patients. This may suggest that PIM1 has independent prognostic value, but larger studies are needed to confirm these findings.

To our knowledge, the role of PIM3 in lung tumors has not been studied so far. Our results reveal abundant PIM3 expression in all BP-NENs, but surprisingly, no higher expression was found in highly aggressive neuroendocrine cancers compared with less aggressive carcinoids. PIM3 mRNA levels were even significantly higher in lung NETs than in lung NECs, and PIM3 protein expression estimated by IHC was quite intense in all BP-NENs, with no significant differences being observed between histopathological groups. No significant relationships were observed between PIM3 mRNA and protein levels among BP-NEN patients. Similar results were obtained for PIM1, although in SCLC, a correlation was noticeable between increased PIM1 mRNA and protein expression. Disjunctions between the levels of mRNA and the protein product are not uncommon: these have been demonstrated in large-scale proteome- and transcriptome-profiling experiments, and this phenomenon is believed to be connected with post-transcriptional, translational, and protein degradation regulation [43, 44].

Our OS analysis results are in line with global statistical data for NET and NEC patients, suggesting that despite its limited size, our study cohort is representative and our results are reliable. Our survival findings are also supported by the presence of a positive correlation between PIM1 and NEK2 expression in BP-NENs. Hence, our results may suggest a coexistence of PIM1 and NEK2 overexpression in SCLC and their contribution to SCLC tumorigenesis. Unfortunately, it was not possible to perform a statistical analysis evaluating the prognostic value of PIM and NEK2 expression in lung NET patients due to no deaths and a small number of relapses in this group. It should be also noted that due to the very low incidence of BP-NENs, especially lung NETs, it is difficult to conduct a large-scale study on BP-NEN patients drawn from only a single center. In addition, as surgery is not a standard therapeutic option in SCLC, adequate primary SCLC specimens for expression studies are difficult to obtain.

To conclude, our study revealed a high expression of NEK2 and PIM kinases in SCLC. Nevertheless, the role of NEK2 and PIM1 overexpression in SCLC tumorigenesis and the clinical relevance of our findings remains unclear. However, data from other cancers indicate that NEK2 and PIM1 kinases possess oncogenic potential and that they interact with diverse downstream signaling pathways. In vitro studies have shown that NEK2 promotes cell proliferation by AKT and Wnt activation in NSCLC [45] and hepatocellular carcinoma [46] and via ERK/MAPK (extracellular signal-regulated kinase/mitogen-activated protein kinase) signaling in gastric cancer [47]. Moreover, NEK2 deregulation was found to act as drivers of tumorigenesis in breast cancer by induction of centrosome amplification [16]. PIM1, in turn, was shown to enhance the growth of lung adenocarcinoma by potentiating the c-MET signaling pathway [41] and the growth of prostate carcinoma [48] and triple-negative breast cancer [49] in cooperation with MYC. Importantly, some reports suggest the involvement of AKT, Wnt, MYC, and MET also in SCLC pathogenesis [50–53]. Hence, further studies are needed to elucidate whether overexpressed NEK2 and PIM1 induce similar promoting effects in SCLC.

Such confirmation is important in the therapeutic context, as NEK2 and PIM kinases have been shown to be potential targets in therapies in different cancers [54, 55]. Indeed, PIM kinase inhibitors, developed as pan-specific ATP binding agents targeting all three kinase isoforms, have undergone clinical trials for hematological malignancies and some solid tumors. Unfortunately, some of these studies were terminated due to compound toxicity and some of them failed to find clinical relevance [55]. More promising results have been obtained from trials combining PIM kinase inhibitors with other oncoprotein inhibitors [55] and a recent in-human study with the PIM447 pan-PIM inhibitor [56]. The development of NEK2 inhibitors is less advanced. Although several NEK2 inhibitors and nucleic acid medicines targeting NEK2 have demonstrated preliminary therapeutic effectiveness against cancer cells both in vitro and in vivo, no NEK2 inhibitors have so far undergone clinical trials [10, 54]. Hence, many questions remain concerning anti-PIM and anti-NEK2 therapies. Even so, they still appear as promising strategies in cancer treatment. In this context, our very preliminary results may serve as a base for further studies leading to the potential use of NEK2 and PIM inhibitors in SCLC therapy. In addition, the fact that PIM3 expression is abundant in all lung neuroendocrine tumors, which was revealed in our research, suggests that this kinase could be potentially a target also in the treatment of other BP-NEN entities.

In conclusion, our data suggests that the aggressive phenotype of BP-NEN is associated with elevated NEK2 and PIM1 expression. Despite being of a preliminary nature, our intriguing findings justify further investigation of the biological function NEK2 and PIM1 in BP-NEN and the translational potential of this knowledge.

## Abbreviations

AC: Atypical carcinoid
AKT: Protein kinase B
BP-NEN: Bronchopulmonary neuroendocrine neoplasm
ERK/MAPK: Extracellular signal-regulated kinase/mitogen-activated protein kinase
FFPE: Formalin-fixed paraffin-embedded
IHC: Immunohistochemistry
JAK/STAT: Janus kinase/signal transducer and activator of transcription
LCNEC: Large cell neuroendocrine carcinoma
mTOR: Mammalian target of rapamycin
NEC: Neuroendocrine carcinoma
NEN: Neuroendocrine neoplasm
NET: Neuroendocrine tumor
NSCLC: Non-small cell lung cancer
OS: Overall survival
SCLC: Small cell lung cancer
TC: Typical carcinoid
WHO: World Health Organization
